# A database on the distribution of butterflies (Lepidoptera) in northern Belgium (Flanders and the Brussels Capital Region)

**DOI:** 10.3897/zookeys.585.8019

**Published:** 2016-04-26

**Authors:** Dirk Maes, Wouter Vanreusel, Marc Herremans, Pieter Vantieghem, Dimitri Brosens, Karin Gielen, Olivier Beck, Hans Van Dyck, Peter Desmet, Vlinderwerkgroep Natuurpunt

**Affiliations:** 1 Research Institute for Nature and Forest (INBO), Kliniekstraat 25, B-1070 Brussels, Belgium; 2 Natuurpunt Studie, Coxiestraat 11, B-2800 Mechelen, Belgium; 3 Vlinderwerkgroep Natuurpunt, Coxiestraat 11, B-2800 Mechelen, Belgium; 4 Leefmilieu Brussel – BIM / Bruxelles Environnement – IBGE, Afd. Groene ruimten, Dpt. Biodiversiteit, Thurn & Taxis-site, Havenlaan 86C/3000 B-1000 Brussels, Belgium; 5 Biodiversity Research Centre, Earth and Life Institute, Université catholique de Louvain, Croix du Sud 4-5, bte L7.07.04, B-1348 Louvain-la-Neuve, Belgium

**Keywords:** Butterflies, distribution, collection, literature, citizen science, observations, monitoring

## Abstract

In this data paper, we describe two datasets derived from two sources, which collectively represent the most complete overview of butterflies in Flanders and the Brussels Capital Region (northern Belgium). The first dataset (further referred to as the *INBO dataset* – http://doi.org/10.15468/njgbmh) contains 761,660 records of 70 species and is compiled by the Research Institute for Nature and Forest (INBO) in cooperation with the Butterfly working group of Natuurpunt (Vlinderwerkgroep). It is derived from the database Vlinderdatabank at the INBO, which consists of (historical) collection and literature data (1830-2001), for which all butterfly specimens in institutional and available personal collections were digitized and all entomological and other relevant publications were checked for butterfly distribution data. It also contains observations and monitoring data for the period 1991-2014. The latter type were collected by a (small) butterfly monitoring network where butterflies were recorded using a standardized protocol. The second dataset (further referred to as the *Natuurpunt dataset* – http://doi.org/10.15468/ezfbee) contains 612,934 records of 63 species and is derived from the database http://waarnemingen.be, hosted at the nature conservation NGO Natuurpunt in collaboration with Stichting Natuurinformatie. This dataset contains butterfly observations by volunteers (citizen scientists), mainly since 2008. Together, these datasets currently contain a total of 1,374,594 records, which are georeferenced using the centroid of their respective 5 × 5 km² Universal Transverse Mercator (UTM) grid cell. Both datasets are published as open data and are available through the Global Biodiversity Information Facility (GBIF).

Research Institute for Nature and Forest

Universal Transverse Mercator

Global Biodiversity Information Facility

## Data published through

INBO dataset: http://doi.org/10.15468/njgbmh

(http://www.gbif.org/dataset/7888f666-f59e-4534-8478-3a10a3bfee45)

Natuurpunt dataset: http://doi.org/10.15468/ezfbee

(http://www.gbif.org/dataset/1f968e89-ca96-4065-91a5-4858e736b5aa)

## Rationale

Butterflies are among the best studied insects in the world and have always attracted the attention of both professional researchers, amateur naturalists, butterfly collectors, and the wider public (Kühn et al. 2008). Butterflies are widely considered as interesting study systems for ecology, evolution, behaviour, and conservation biology (e.g., Watt and Boggs 2003). Many butterflies have been collected and subsequently stored in museum or private collections. Furthermore, entomologists have often published lists of observed species during excursions to special habitats or have made overviews of regional or national butterfly faunas. In Belgium, entomology in general and lepidopterology in particular, have a long tradition with the first faunas already published only seven years after its independence in 1830 (De Selys-Longchamps 1837). Since then, several authors have updated the Belgian butterfly fauna based on collections or observations (e.g., Hackray et al. 1969; De Prins 1998). In 1991, the youth and nature organization Jeugdbond voor Natuur en Milieu (JNM) launched a butterfly project with the aim to publish a distribution atlas of the butterflies of Flanders, northern Belgium (Daniëls 1991). To do so, a first step consisted of collecting all historical collection and literature data. Secondly, a working group was organised in cooperation between JNM, De Wielewaal (which later became Natuurpunt) and the INBO that set up a citizen science project to obtain as many butterfly observations with a good spatial coverage over Flanders. The data gathered during this project (period 1991-1998) were used to compile a first Red List (Maes and Van Dyck 2001) and a distribution atlas of butterflies in Flanders, including the Brussels Capital Region (Maes and Van Dyck 1999). Recently, both the Red List (Maes et al. 2012) and the distribution atlas (Maes et al. 2013) were updated using recent distribution data recorded through www.waarnemingen.be, a data portal launched by Natuurpunt, the largest nature conservation NGO in Belgium, where citizen-scientists can store and keep track of their recordings. Here, we publish both the historical and the more recent data used for the Red List and the distribution atlases as a data paper on a UTM grid cell resolution of 5 × 5 km².

## Taxonomic coverage

The datasets cover all 67 indigenous and 3 regular migrant butterfly species (*Colias croceus*, *Colias hyale*, *Vanessa cardui*). In the INBO dataset vagrant or doubtful species (*Apatura ilia*, *Arethusana arethusa*, *Boloria dia*, *Brenthis ino*, *Coenonympha arcania*, *Colias alfacariensis*, *Colias palaeno*, *Cupido argiades*, *Danaus plexippus*, *Erebia aethiops*, *Erebia ligea*, *Erebia medusa*, *Hamearis lucina*, *Iphiclides podalirius*, *Lampides boeticus*, *Lasiommata maera*, *Limenitis populi*, *Limenitis reducta*, *Lycaena dispar*, *Lycaena helle*, *Lycaena hippothoe*, *Lycaena virgaureae*, *Melitaea aurelia*, *Pontia daplidice*) and introduced species (*Cacyreus marshalli* and *Polyommatus damon*) were excluded because no evidence of the observation was available. In the Natuurpunt dataset, however, eight vagrant species with photographic evidence, that most likely spontaneously reached Flanders were included (*Apatura ilia*, *Brenthis ino*, *Cupido argiades*, *Iphiclides podalirius*, *Lampides boeticus*, *Nymphalis xanthomelas*, *Polyommatus coridon* and *Pontia daplidice*). Three additional species (*Aporia crataegi*, *Argynnis adippe* and *Argynnis aglaja*) are considered as indigenous species, but recent observations are all vagrant individuals. Nomenclature is according to Fauna Europaea (http://www.faunaeur.org/full_results.php?id=7).

## Taxonomic ranks

**Kingdom**: Animalia

**Phylum**: Arthropoda, **Subphylum**: Hexapoda, **Class**: Insecta, **Order**: Lepidoptera, **Superfamilies**: Hesperoidea, Papilioidea, **Families**: Hesperiidae, Lycaenidae, Nymphalidae, Papilionidae, Pieridae, **Subfamilies**: Apaturinae, Coliadinae, Dismorphiinae, Heliconiinae, Heteropterinae, Hesperiinae, Limenitidinae, Lycaeninae, Melitaeinae, Nymphalinae, Papilioninae, Pierini, Polyommatinae, Pyrginae, Satyrinae, Theclinae, **Genera**: *Aglais*, *Anthocharis*, *Apatura*, *Aphantopus*, *Aporia*, *Araschnia*, *Argynnis*, *Aricia*, *Boloria*, *Callophrys*, *Carcharodus*, *Carterocephalus*, *Celastrina*, *Coenonympha*, *Cupido*, *Cyaniris*, *Erynnis*, *Euphydryas*, *Favonius*, *Gonepteryx*, *Hesperia*, *Heteropterus*, *Hipparchia*, *Issoria*, *Lasiommata*, *Leptidea*, *Limenitis*, *Lycaena*, *Maniola*, *Melitaea*, *Melanargia*, *Nymphalis*, *Ochlodes*, *Papilio*, *Pararge*, *Phengaris*, *Pieris*, *Plebejus*, *Polygonia*, *Polyommatus*, *Pyrgus*, *Pyronia*, *Satyrium*, *Spialia*, *Thecla*, *Thymelicus*, *Vanessa*

**Species**: Table 1 gives an overview of the species, together with the number of records present in the respective datasets.

**Table 1.** The number of records per species in the two datasets and the sum of the records in both datasets. v = observations with photographic evidence, but the species most probably do not have populations in Flanders. ^†^ indicates that a species is considered as extinct in Flanders; the year of extinction is also given. Observations after the year of extinction are considered as vagrant individuals. ^M^: regular migrant species, ^(M)^: the species is indigenous, but the regional population is supplemented by migrant individuals.

Species name	INBO	Natuurpunt	Total
*Aglais io*	54,329	52,471	106,800
*Aglais urticae*	35,237	25,047	60,284
*Anthocharis cardamines*	15,689	17,393	33,082
*Apatura ilia*	-	4^v^	4
*Apatura iris*	141	304	445
*Aphantopus hyperantus*	8,156	7,636	15,792
*Aporia crataegi* ^†1960^	120	2^v^	122
*Araschnia levana*	24,772	18,531	43,303
*Argynnis adippe* ^†1947^	22	3^v^	25
*Argynnis aglaja* ^†1971^	54	1^v^	55
*Argynnis niobe* ^†1977^	21	-	21
*Argynnis paphia*	272	697	969
*Aricia agestis*	6,867	5,251	12,118
*Boloria euphrosyne* ^†1949^	37	-	37
*Boloria selene* ^†1994^	181	-	181
*Brenthis ino*	-	7^v^	7
*Callophrys rubi*	2,008	1,552	3,560
*Carcharodus alceae*	16	402	418
*Carterocephalus palaemon*	1,159	2,478	3,637
*Celastrina argiolus*	21,857	20,579	42,436
*Coenonympha hero* ^†1912^	16	-	16
*Coenonympha pamphilus*	9,886	10,429	20,315
*Coenonympha tullia* ^†1994^	70	-	70
*Colias croceus* ^M^	3,380	12,762	16,142
*Colias hyale* ^M^	617	277	894
*Cupido argiades*	-	1^v^	1
*Cupido minimus*	82	43	125
*Cyaniris semiargus*	222	76	298
*Erynnis tages*	102	130	232
*Euphydryas aurinia* ^†1959^	65	-	65
*Favonius quercus*	2,217	3,051	5,268
*Gonepteryx rhamni*	20,011	22,357	42,368
*Hesperia comma*	145	471	616
*Heteropterus morpheus* ^†1995^	29	-	29
*Hipparchia semele*	4,157	5,160	9,317
*Hipparchia statilinus* ^†1930^	11	-	11
*Iphiclides podalirius*	-	5^v^	5
*Issoria lathonia*	2,794	3,216	6,010
*Lampides boeticus*	-	44^v^	44
*Lasiommata megera*	4,089	1,882	5,971
*Leptidea sinapis*	144	585	729
*Limenitis camilla*	1,154	2,323	3,477
*Limenitis populi* ^†1957^	14	-	14
*Lycaena phlaeas*	16,393	15,246	31,639
*Lycaena tityrus*	303	263	566
*Maniola jurtina*	35,117	31,782	66,899
*Melanargia galathea*	53	23	76
*Melitaea athalia* ^†1968^	80	-	80
*Melitaea cinxia*	300	466	766
*Melitaea diamina* ^†1954^	28	-	28
*Nymphalis antiopa*	240	63	303
*Nymphalis polychloros*	323	362	685
*Nymphalis xanthomelas*	-	5^v^	5
*Ochlodes sylvanus*	11,484	15,660	27,144
*Papilio machaon*	10,322	8,927	19,249
*Pararge aegeria*	65,290	56,129	121,419
*Phengaris alcon*	441	342	783
*Phengaris teleius* ^†1980^	136	-	136
*Pieris brassicae*	45,713	22,030	67,743
*Pieris napi*	54,313	28,294	82,607
*Pieris rapae*	94,957	52,188	147,145
*Plebejus argus*	1,436	1,711	3,147
*Plebejus idas* ^†1984^	15	-	15
*Polygonia c-album*	33,660	36,058	69,718
*Polyommatus coridon*	-	12^v^	12
*Polyommatus icarus*	20,269	21,186	41,455
*Pontia daplidice*	-	3^v^	3
*Pyrgus armoricanus* ^†1952^	18	-	18
*Pyrgus malvae*	589	527	1116
*Pyronia tithonus*	31,771	21,184	52,955
*Satyrium ilicis*	397	617	1,014
*Satyrium w-album*	97	504	601
*Spialia sertorius* ^†1937^	8	-	8
*Thecla betulae*	835	2,191	3,026
*Thymelicus lineola*	17,087	5,029	22,116
*Thymelicus sylvestris*	1,012	387	1,399
*Vanessa atalanta* ^(*M*)^	69,965	55,306	125,271
*Vanessa cardui* ^M^	28,865	21,269	50,134
Total	761,660	612,934	1,374,594
N species	70	63	78
Number of grid cells surveyed	631	634	637
Number of different observers	1,697	3,856	

**Common names**: Butterflies

## Geographic coverage

### Flanders and the Brussels Capital Region

**Figure 1.**
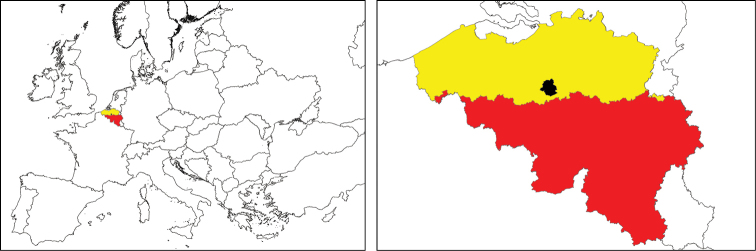
The location of Belgium in Europe (left) and the three administrative regions of Belgium (right): Flanders (yellow), the Brussels Capital Region (black) and Wallonia (red).

Flanders and the Brussels Capital Region cover an area of 13,522 km² and 162 km² respectively (13,684 km² in total – Figure 1). This area is situated in the northern of Belgium and represents 45% of the Belgian territory. Flanders is largely covered by agricultural land and urban areas while the Brussels Capital Region is mainly urban (Table 2). Both regions have a very high population density (Table 2).

**Table 2.** Area of the main land use types in Flanders and the Brussels Capital Region in ha (ranked in descending order of percentage in both regions). Source: Biological Valuation Map Flanders and the Brussels Capital Region (Vriens et al. 2011).

Land use type	Flanders	Land use type	Brussels Capital Region
Agricultural land	702 276 (51%)	Urban areas	11 917 (73%)
Urban areas	411 144 (30%)	Woodlands	1988 (12%)
Woodlands	138 595 (10%)	Other green areas	1568 (10%)
Other green areas	39 516 (3%)	Agricultural land	544 (3%)
Water	32 008 (2%)	Water	185 (1%)
Semi-natural grasslands	15 315 (1%)	Semi-natural grasslands	27 (<1%)
Heathlands	8140 (<1%)	Marshes	17 (<1%)
Coastal dunes	1818 (<1%)	Heathlands	3 (<1%)
Marshes	1742 (<1%)		
Mud flats and salt marshes	1497 (<1%)		
Population density	474/km²		7210/km²

## Bounding box

50°40'48"N to 51°30'36"N latitude, 2°32'24"E to 5°55'12"E longitude

## Georeferencing method

**Figure 2.**
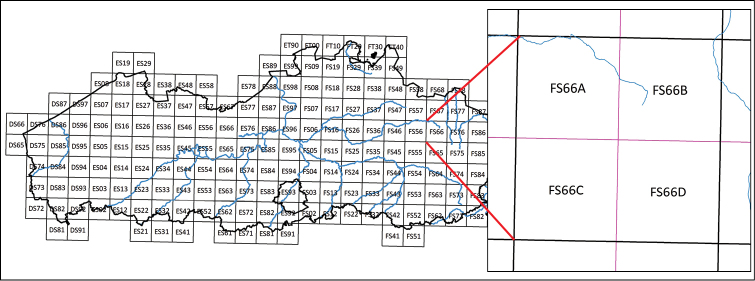
10 × 10 km² UTM grid cells in Flanders and in the Brussels Capital Region. The partitioning of 10 × 10 km² UTM grid cells (left) into 5 × 5 km² UTM grid cells is shown on the right. The 5 × 5 km² UTM grid cells were used to georeference the distribution data in Flanders and the Brussels Capital Region.

All distribution data of butterflies in Flanders and the Brussels Capital Region were attributed to grid cells of 5 × 5 km² of the Universal Transverse Mercator (UTM) projection (Figure 2). The centroids of the 5 × 5 km² grid cells were calculated using the WGS84 projection with a *coordinateUncertaintyInMeters* of 3,769 meters (Wieczorek et al. 2004).

In total, Flanders and the Brussels Capital Region cover 638 (622 with records) and 9 (all nine with records) grid cells, respectively. The grid cells without records only cover a very small area within Flanders.

## Temporal coverage

**Figure 3.**
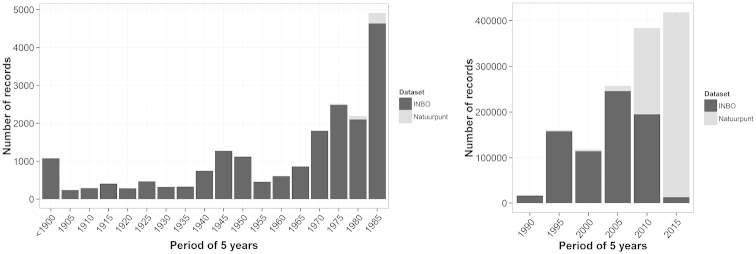
Number of collected records between 1830 and 1985 (left) and between 1986 and 2014 (right) in the two datasets (INBO and Natuurpunt). Each number on the x-axis stands for a period of 5 years (e.g., 1905 = 1901–1905, 1910 = 1906–1910, etc.). Note the different scales on the y-axis for both figures.

The INBO dataset mainly covers the historical museum and literature records (since 1830), butterfly monitoring records (since 1991) and observations (until 2008) while the Natuurpunt dataset covers the recent observations (mostly since 2008). Between 2000 and 2006, a butterfly survey project was organised in the province of West-Flanders (Cuvelier et al. 2007) and in the period 2006-2008, a similar project was undertaken in the Brussels Capital Region by the INBO on demand of Leefmilieu Brussel – BIM (Beckers et al. 2009). Both datasources were integrated in the INBO dataset. Since the introduction of the data portal www.waarnemingen.be for storing observations by the NGO Natuurpunt in 2008, the number of records has strongly increased and now reaches almost 150,000 records per year (Figure 3). The datasets will be updated on a yearly basis.

## Methodology

### Sampling methods

Butterfly distribution data were collected in four different ways: i) collection data, ii) literature data, iii) monitoring transect data and iv) observations.

Collection data were digitized from the following museum collections: Bosmuseum Groenendaal, Royal Institute for Natural Sciences (Brussels), Agricultural Faculty of Gembloux, Ghent university and the Antwerp Zoo. Furthermore, the private butterfly collections of the following people were also incorporated into the INBO dataset: A. Artoisenet, R. Bracke, A. Caljon, S. Cuvelier, A. De Boer, K. Desender, P. Halflants, D. Hilven, J. & T. Jaeken, M. Keirens, H. Kinders, P. & W. Pardon, W. Tips, W. Troukens, F. Turelinckx, O. Van De Kerckhove, R. Van Heuverswijn, B. Vandepitte, J. Vervaeke & R. Winnen. The source collection is indicated in the field *associatedReferences*.

Published observations were searched for in different literature sources (see section “References to literature checked for occurrence data” in the Suppl. material 1) and indicated in the field *associatedReferences*. Since most of the records in collections and in the literature were only reported at the municipality level, the UTM 5 × 5 km² grid cell of the centre of the municipality was attributed to the record.

Butterfly monitoring counts were conducted along fixed transects of maximum 1 km, consisting of smaller sections, each with a homogeneous habitat (e.g., woodland, hay meadow, dry heathland – see van Swaay et al. 2008; van Swaay et al. 2011 for a detailed description of the monitoring method).

**Figure 4.**
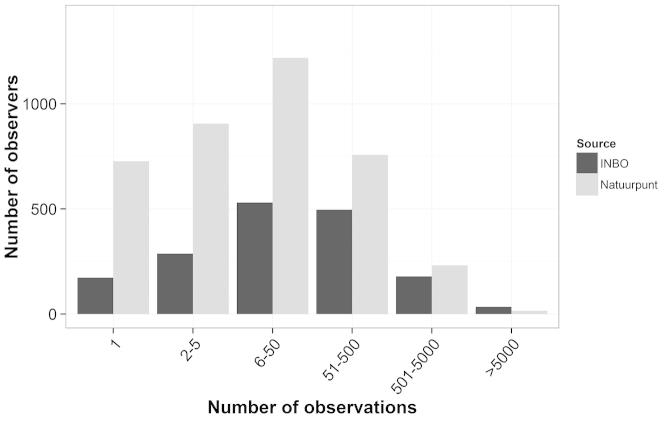
Frequency distribution of the observers per number of records in the datasets of INBO and Natuurpunt.

Observations (species, date, location, observer) were recorded by volunteers/citizen scientists and stored in the INBO dataset (mainly for the period 1991-2007, usually with a resolution of 1 × 1 km² or 5 × 5 km²) or in the Natuurpunt dataset. Since 2011, 69% of the records had a precision of 25m or less. Because of the increasing popularity of mobile apps using GPS readings in the field, this proportion increased with 5% per year to reach 77% in 2015. The number of observers in the INBO and the Natuurpunt datasets is given in Table 1. The frequency distribution of the recorders per number of records is given in Figure 4.

A list of references that used data described in this paper can be found in the section “Publications based on this dataset” in the Suppl. material 1.

### Quality control

The data in both datasets were carefully verified by butterfly experts (including professional entomologists) taking collection specimens, the observer’s species knowledge, added photographs and known species list of locations into account. The validation procedure from www.waarnemingen.be consists of an interactive procedure in which observers can be asked for additional information by a team of validators, after which the validator manually adds a validation status. Records that are not manually validated are additionally checked by an automated validation procedure that takes into account the number of manually validated observations within a specified date and distance range. 11% of the butterfly records submitted to the data portal www.waarnemingen.be are supported by photographs. The validation status is indicated in the field *identificationVerificationStatus*.

### Information withheld

In the original databases, the observer’s name, the exact XY-coordinates and the toponym are known.

## Datasets

### Dataset description

The butterfly occurrence data are published as two separate Darwin Core Archives: 1) collection and literature data, observations and butterfly monitoring in Flanders and in the Brussels Capital Region (1830-2014) hosted at the Research Institute for Nature and Forest (INBO) and 2) recent observations (1974-2014) from the Natuurpunt data portal (www.waarnemingen.be). The data models used for both datasets are identical and can be merged easily. The INBO dataset contains 761,660 records and the Natuurpunt dataset 612,934 records totalling to almost 1.4 million records. The data compiled for the butterfly atlas of the Brussels Capital Region are marked as INBO/LB-BIM in the *ownerInstitutionCode* field in the INBO dataset.

**Figure 5.**
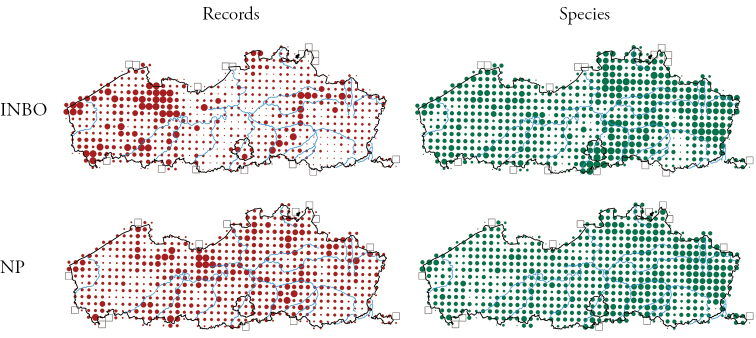
Number of records (left, increasing dot sizes represent 100, 1000, 2500, 5000 and >5000 records per grid cell) and species (right, increasing dot sizes represent 10, 20, 30, 40 and >40 species per grid cell) in the INBO dataset (1830–2014, top row) and in the NP dataset (1981–2014, bottom row). Squares indicate grid cells without records.

The distribution of the number of records and species per grid cell for both datasets is given in Figure 5.

The data are standardized to Darwin Core (Wieczorek et al. 2012) with a custom SQL view on the original INBO and Natuurpunt butterfly database respectively. They were published using the GBIF Integrated Publishing Toolkit (Robertson et al. 2014) instance at the INBO (http://data.inbo.be/ipt). The Darwin Core terms (http://rs.tdwg.org/dwc/terms/) in the dataset at the time of publication are:

*occurrenceID*, *type*, *language*, *license*, *rightsHolder*, *accessRights*, *references*, *datasetID*, *institutionCode*, *datasetName*, *ownerInstitutionCode*, *basisOfRecord*, *informationWithheld*, *dataGeneralizations*, *recordedBy*, *individualCount*, *sex*, *lifestage*, *associatedReferences*, *samplingProtocol*, *samplingEffort*, *eventDate*, *verbatimEventDate*, *continent*, *countryCode*, *stateProvince*, *municipality*, *verbatimLocality*, *verbatimCoordinates*, *verbatimCoordinateSystem*, *verbatimSRS*, *decimalLatitude*, *decimalLongitude*, *geodeticDatum*, *coordinateUncertaintyInMeters*, *georeferenceRemarks*, *identificationVerificationStatus*, *scientificName*, *kingdom*, *phylum*, *class*, *order*, *taxonRank*, *scientificNameAuthorship*, *vernacularName*, *nomenclaturalCode*.

### INBO dataset

• **Object name**: Vlinderdatabank – Butterflies in Flanders and the Brussels Capital Region, Belgium

• **Format name**: Darwin Core Archive format

• **Format version**: 1.0

• **Character encoding**: UTF-8

• **Language**: English

• **License**: http://creativecommons.org/publicdomain/zero/1.0/

• **Usage norms**: http://www.inbo.be/en/norms-for-data-use

• **Publication date**: 2016-01-13

• **Distribution**: http://dataset.inbo.be/dagvlinders-inbo-occurrences

• **DOI**: http://doi.org/10.15468/njgbmh

### Natuurpunt dataset

• **Object name**: Waarnemingen.be – Butterfly observations in Flanders and the Brussels Capital Region, Belgium

• **Format name**: Darwin Core Archive format

• **Format version**: 1.0

• **Character encoding**: UTF-8

• **Language**: English

• **License**: http://creativecommons.org/publicdomain/zero/1.0/

• **Usage norms**: http://www.natuurpunt.be/normen-voor-datagebruik

• **Publication date**: 2016-02-02

• **Distribution**: http://dataset.inbo.be/dagvlinders-natuurpunt-occurrences

• **DOI**: http://doi.org/10.15468/ezfbee

### Usage norms

To allow anyone to use the datasets described here, we released the data to the public domain under a Creative Commons Zero waiver (http://creativecommons.org/publicdomain/zero/1.0/). Users of published datasets are encouraged to follow the respective norms for data use (http://www.inbo.be/en/norms-for-data-use and http://www.natuurpunt.be/normen-voor-datagebruik [in Dutch]) and to provide a link to the original dataset (http://doi.org/10.15468/njgbmh and http://doi.org/10.15468/ezfbee), whenever appropriate. If used for a scientific paper, it is recommended to cite the dataset following the applicable citation norms (e.g. GBIF 2012) and/or to contact the authors for additional information (dirk.maes@inbo.be, marc.herremans@natuurpunt.be or dimitri.brosens@inbo.be). Dataset issues can also be reported via opendata@inbo.be.

## Supplementary materials

Supplementary material 1 A database on the distribution of butterflies (Lepidoptera) in northern Belgium (Flanders and the Brussels Capital Region) Data type: Additional references Explanation note: The supplementery material provides 1) a list of published entomological references that were checked for occurrence data and 2) a list of references that used data described in this paper. This dataset is made available under the Open Database License (http://opendatacommons.org/licenses/odbl/1.0/). The Open Database License (ODbL) is a license agreement intended to allow users to freely share, modify, and use this Dataset while maintaining this same freedom for others, provided that the original source and author(s) are credited.Dirk Maes, Wouter Vanreusel, Marc Herremans, Pieter Vantieghem, Dimitri Brosens, Karin Gielen, Olivier Beck, Hans Van Dyck, Peter Desmet, Vlinderwerkgroep Natuurpunt
