# Supplementary material for: A database on the distribution of butterflies (Lepidoptera) in northern Belgium (Flanders and the Brussels Capital Region)
Source: Zookeys. 2016 Apr 26;(585):143–56. doi: 10.3897/zookeys.585.8019 (PMC4857040; doi:10.3897/zookeys.585.8019)
Supplement: Supplementary material 1 — A database on the distribution of butterflies (Lepidoptera) in northern Belgium (Flanders and the Brussels Capital Region) [file zookeys-585-143-s001.docx]

Supplementary Material

A database on the distribution of butterflies (Lepidoptera) in northern Belgium (Flanders and the Brussels Capital Region)

Dirk Maes^1^, Wouter Vanreusel^2^, Marc Herremans^2^, Pieter Vantieghem^3^, Dimitri Brosens^1^, Karin Gielen^2^, Olivier Beck^4^, Hans Van Dyck^5^, Peter Desmet^1^ & Vlinderwerkgroep Natuurpunt^3^

References to literature checked for occurrence data (chronological order)

### Books and catalogues

De Selys-Longchamps, E (1837). Catalogue des Lepidoptères ou Papillons de la Belgique. Luik. pp. 14-21

De Selys-Longchamps, E (1844). Enumération des insectes Lépidoptères de la Belgique.

Quaedvlieg, L (1873). Les Papillons diurnes de Belgique, manuel du jeune lépidoptèrologiste.

Dubois, CF, Dubois, A (1874). Les Lépidoptères de la Belgique.

Lambillion, LJ (1900). Catalogue des Lépidoptères de Belgique. Imprimerie Douxfils, Namur.

Lambillion, LJ (1903). Catalogue des Lépidoptères de Belgique. Imprimerie Douxfils, Namur.

Lambillion, LJ (1907). Catalogue des Lépidoptères de Belgique, Addenda. Imprimerie Douxfils, Namur.

Verstraeten, C (1970). Atlas provisoire des insectes de Belgique, cartes 187-200. Gembloux.

Verstraeten, C (1971). Atlas provisoire des insectes de Belgique, cartes 551-582. Gembloux.

Verstraeten, C, De Prins, W (1976). Atlas provisoire des insectes de Belgique, cartes 965-1000. Gembloux.

Janssen, A (1983). De dagvlinders van de Belgische fauna, Vereniging voor Entomologie van de Koninklijke Maatschappij voor Dierkunde van Antwerpen, Antwerpen.

Verstraeten, C (1985). Atlas provisoire des insectes de Belgique, cartes 1867-1891. Gembloux.

De Tré, E (1987). Entomobrochure 5: Inventarisatie, status en ekologie van het Belgisch dagvlinderbestand, Vlaamse Vereniging voor Entomologie, Antwerpen.

Taymans, C, De Bast, B, Verstraeten, C, Baguette, M, Goffart, P (1992). Mise jour de la cartographie des Rhopalocères de la Belgique: genre *Pyrgus* Hübner, 1819 (Lepidoptera, Hesperiidae). In: Van Goethem JL, Grootaert P (eds.) Proceedings of the 8th International Colloquium of the European Invertebrate Survey Faunal inventories of sites for cartography and nature conservation. KBIN, Brussel, 173-176.

Vanholder, B, De Turck, A, Glabeke, G, Misonne, B, Troukens, W, Van Opstaele, M, Vermandel, E (1995). De Belgische trekvlinders en dwaalgasten (10 jaar Belgisch trekvlinderonderzoek), Vlaamse Vereniging voor Entomologie, Antwerpen.

De Prins, W (1998). Catalogue of the Lepidoptera of Belgium, Koninklijk Belgisch Instituut voor Natuurwetenschappen, Brussel.

Cuvelier, S, Degrande, J, Merveillie, L, Spruytte, S, Vervaeke, J (2007). Dagvlinders in West-Vlaanderen. Verspreiding en Ecologie 2000-2006, Z.W.V.V.K., Ieper, 144 pp.

### Local and regional journals

Atalanta, Biologisch Jaarboek Dodonaea, Bulletin & Annales de la Société royale belge d'Entomologie, Euglena, Koerier, Lambillionea, Linneana Belgica, Natuur en Landschap, Phegea, Revue Mensuelle de la Société Entomologique Namuroise, Schakel, Stentor, Vlinders, Wielewaal, Wielewaaltje.

## Publications based on this dataset (chronological order)

### International (A1) publications

Maes, D, van Swaay, CAM (1997). A new methodology for compiling national Red Lists applied on butterflies (Lepidoptera, Rhopalocera) in Flanders (N.-Belgium) and in The Netherlands. Journal of Insect Conservation, 1: 113-124. doi: 10.1023/A:1018435110335

van Swaay, CAM, Maes, D, Plate, C (1997) Monitoring butterflies in The Netherlands and Flanders: the first results. Journal of Insect Conservation, 1: 81-88. doi: 10.1023/A:1018491228082

Maes, D, Van Dyck, H (2001). Butterfly diversity loss in Flanders (north Belgium): Europe's worst case scenario? Biological Conservation, 99: 263-276. doi: 10.1016/S0006-3207(00)00182-8

Maes, D, Gilbert, M, Titeux, N, Goffart, P, Dennis, RLH (2003). Prediction of butterfly diversity hotspots in Belgium: a comparison of statistically focused and land use-focused models. Journal of Biogeography, 30: 1907-1920. doi: 10.1046/j.0305-0270.2003.00976.x

Maes, D, Bauwens, D, De Bruyn, L, Anselin, A, Vermeersch, G, Van Landuyt, W, De Knijf, G, Gilbert, M (2005). Species richness coincidence: conservation strategies based on predictive modelling. Biodiversity and Conservation, 14: 1345-1364. doi: 10.1007/s10531-004-9662-x

Maes, D, Anselin, A, van Swaay, CAM, Sierdsema, H, De Bruyn, L, Vermeersch, G, Van Dyck, H (2009). Can we predict the distribution of heathland butterflies with heathland bird data? Animal Biology, 59: 335-349. doi: 10.1163/157075609x454962

Titeux, N, Maes, D, Marmion, M, Luoto, M, Heikkinen, RK (2009). Inclusion of soil data improves the performance of bioclimatic envelope models for insect species distributions in temperate Europe. Journal of Biogeography, 36: 1459-1473. doi: 10.1111/j.1365-2699.2009.02088.x

Van Dyck, H, van Strien, AJ, Maes, D, van Swaay, CAM (2009). Declines in common, widespread butterflies in a landscape under intense human use. Conservation Biology, 23: 957-965. doi: 10.1111/j.1523-1739.2009.01175.x

Maes, D, Titeux, N, Hortal, J, Anselin, A, Decleer, K, De Knijf, G, Fichefet, V, Luoto, M (2010). Predicted insect diversity declines under climate change in an already impoverished region. Journal of Insect Conservation, 14: 485-498. doi: 10.1007/s10841-010-9277-3

van Swaay, CAM, Maes, D, Collins, S, Munguira, ML, Šašić, M, Settele, J, Verovnik, R, Warren, MS, Wiemers, M, Wynhoff, I, Cuttelod, A (2011). Applying IUCN criteria to invertebrates: How red is the Red List of European butterflies? Biological Conservation, 144: 470-478. doi: 10.1016/j.biocon.2010.09.034

Maes, D, Vanreusel, W, Jacobs, I, Berwaerts, K, Van Dyck, H (2012). Applying IUCN Red List criteria at a small regional level: A test case with butterflies in Flanders (north Belgium). Biological Conservation, 145: 258-266. doi: 10.1016/j.biocon.2011.11.021

Carvalheiro, LG, Kunin, WE, Keil, P, Aguirre-Gutiérrez, J, Ellis, WN, Fox, R, Groom, QJ, Hennekens, SM, Van Landuyt, W, Maes, D, Van de Meutter, F, Michez, D, Rasmont, P, Odé, B, Potts, SG, Reemer, M, Masson Roberts, SP, Schaminée, JHJ, WallisdeVries, MF, Biesmeijer, JC (2013). Biodiversity declines and biotic homogenization have slowed for NW Europe pollinators and plants. Ecology Letters, 16: 870-878. doi: 10.1111/ele.12121

Maes, D, Jacobs, I, Segers, N, Vanreusel, W, Van Daele, T, Laurijssens, G, Van Dyck, H (2014). A resource-based conservation approach for an endangered ecotone species: the Ilex Hairstreak (*Satyrium ilicis*) in Flanders (north Belgium). Journal of Insect Conservation, 18: 939-950. doi: 10.1007/s10841-014-9702-0

Maes, D, Isaac, NB, Harrower, C, Collen, B, van Strien, A, Roy, DB (2015). The use of opportunistic data for IUCN Red List assessments. Biological Journal of the Linnean Society, 115: 690-706. doi: 10.1111/bij.12530

Van Dyck, H, Puls, R, Bonte, D, Gotthard, K, Maes, D (2015). The lost generation hypothesis: could climate change drive ectotherms into a developmental trap? Oikos, 124: 54-61. doi: 10.1111/oik.02066

### International reports

van Swaay, CAM, van Strien, AJ, Harpke, A, Fontaine, B, Stefanescu, C, Roy, DB, Maes, D, Kühn, E, Õunap, E, Regan, E, Švitra, G, Heliölä, J, Settele, J, Warren, MS, Plattner, M, Kuussaari, M, Cornish, N, Garcia Pereira, P, Leopold, P, Feldmann, R, Julliard, R, Verovnik, R, Popov, S, Brereton, TM, Gmelig Meyling, AW, Collins, S (2010). The European Butterfly Indicator for Grassland species 1990-2009, Report De Vlinderstichting, Wageningen.

van Swaay, CAM, van Strien, AJ, Harpke, A, Fontaine, B, Stefanescu, C, Roy, D, Maes, D, Kühn, E, Õunap, E, Regan, E, Švitra, G, Prokofev, I, Heliölä, J, Settele, J, Pettersson, LB, Botham, M, Musche, M, Titeux, N, Cornish, N, Leopold, P, Julliard, R, Verovnik, R, Öberg, S, Popov, S, Collins, S, Goloshchapova, S, Roth, T, Brereton, T, Warren, MS (2013). The European Grassland Butterfly Indicator 1990-2011, EEA Technical report No 11/2013. European Environmental Agency, Copenhagen, Denmark, 36 pp.

van Swaay, CAM, van Strien, AJ, Aghababyan, K, Åström, S, Botham, M, Brereton, T, Chambers, P, Collins, S, Domènech Ferrés, M, Escobés, R, Feldmann, R, Fernández-García, J-M, Fontaine, B, Goloshchapova, S, Gracianteparaluceta, A, Harpke, A, Heliölä, J, Khanamirian, G, Julliard, R, Kühn, E, Lang, A, Leopold, P, Loos, J, Maes, D, Mestdagh, X, Monasterio, Y, Munguira, ML, Murray, T, Musche, M, Õunap, E, Pettersson, L, Popoff, S, Prokotev, S, Roth, T, Roy, DB, Settele, J, Stefanescu, C, Švitra, G, Marques Teixeira, S, Tiitsaar, A, Verovnik, R, Warren, MS (2015). The European butterfly Indicator for Grassland species: 1990-2013, Rapport VS2015.009. De Vlinderstichting, Wageningen, 37 pp.

### Local publications

Daniëls, L, De Tré, E, Wittoeck, J, Vromant, C, Maes, D (1992). JNM-Dagvlinderinventarisatie: eerste gedeeltelijke resultaten, Jeugdbond voor Natuurstudie en Milieubescherming, Gent.

Maes, D, Daniëls, L (1993). Voorlopige atlas van de Vlaamse dagvlinders. Euglena, 12: 1-65.

Veling, K, Daniëls, L, Maes, D (1993). Opvallende waarnemingen 1992 in Nederland en Vlaanderen. Vlinders, 8: 4-6.

Maes, D, Daniëls, L (1994). Dagvlinders in Limburg: vroeger en nu. In: Likona (ed.) Jaarboek LIKONA 1993. Limburgse Koepel voor Natuurstudie, Hasselt, 32-40.

Raes, D, Maes, D (1995). In het Zoniënbos hebben vlinders een toekomst! Vlinders, 10: 4-6.

Van Dyck, H, Maes, D (1995). De parel van het schrale veld. Vlinders, 10: 7-9.

Maes, D (1996). De Iepepage in Vlaanderen: terug van (nooit) weg geweest? Vlinders, 11: 4-6.

Maes, D, Van Dyck, H (1996). De Rode lijst dagvlinders van Vlaanderen. Vlinders, 11: 21-23.

Maes, D, Van Dyck, H (1996). Een gedocumenteerde Rode lijst van de dagvlinders van Vlaanderen, Mededelingen van het Instituut voor Natuurbehoud Instituut voor Natuurbehoud, Brussel, 154 pp.

van Swaay, CAM, Plate, C, Maes, D (1996). Vijf jaar dagvlindermonitoring in Nederland en Vlaanderen. Vlinders, 11: 22-26.

Maes, D (1997). Het gebruik van vlindergegevens in het natuurbehoud in Vlaanderen. De Levende Natuur, 98: 189-194.

Maes, D, Van Dyck, H (1997). Een Zilveren-maansverduistering in Vlaanderen. Vlinders, 12: 8-11.

Maes, D, Van Dyck, H (1999). Dagvlinders in Vlaanderen - Ecologie, verspreiding en behoud, Stichting Leefmilieu i.s.m. Instituut voor Natuurbehoud en Vlaamse Vlinderwerkgroep, Antwerpen/Brussel, 480 pp.

Maes, D, Van Dyck, H (1999). Dagvlinders. In: Kuijken E (ed.) Natuurrapport 1999. Toestand van de natuur in Vlaanderen: cijfers voor het beleid. Instituut voor Natuurbehoud, Brusse, 73-77.

Berwaerts, K, Maes, D, Meyermans, F, Gorissen, D (2000). Vergane glorie van het Walenbos? Vlinders, 15: 24-27.

Bauwens, D, Maes, D, De Knijf, G, Anselin, A (2001). Criteria voor het aanwijzen van prioritaire soorten voor het natuurbeleid in de provincie Antwerpen, Instituut voor Natuurbehoud, Brussel.

Bauwens, D, Maes, D, De Knijf, G, Anselin, A (2001). Criteria voor het aanwijzen van prioritaire soorten voor het natuurbeleid in de provincie Limburg, Instituut voor Natuurbehoud, Brussel.

Maes, D, Van Dyck, H (2001). Dagvlinders in Limburg. In: Likona (ed.). Limburgse Koepel voor Natuurstudie, Genk, 73-77.

Maes, D, Van Dyck, H (2002). Veranderingen in het dagvlinderbestand in Antwerpen: lessen voor het natuurbeleid! ANKONA-jaarboek. Antwerpse Koepel voor Natuurstudie, Antwerpen, 43-56.

Verschraegen, T, Vanreusel, W, Lambrechts, J, Maes, D (2003). Klavertje vier. Het bruin dikkopje, het boswitje, het dwergblauwtje en het klaverblauwtje in Vlaanderen. Vlinders, 18: 4-6.

Gorissen, D, Merckx, T, Vercoutere, B, Maes, D (2004). Veranderd bosgebruik en dagvlinders. Waarom verdwenen dagvlinders uit bossen in Vlaanderen? Landschap, 21: 85-95.

Maes, D, Bonte, D, Broidioi, J (2004). Dagvlinders. In: Provoost S, Bonte D (eds.) Levende duinen: een overzicht van de biodiversiteit aan de Vlaamse kust. Instituut voor Natuurbehoud, Brussel, 272-285.

Verheyen, K, Piessens, K, Desender, K, Van Dyck, H, Van Elegem, B, Vermeersch, G, Van Landuyt, W, Maes, D (2005). Veranderingen in biodiversiteit van bos en heide door de eeuwen heen. Relaties tussen flora, fauna en landschapsdynamiek. Natuur.focus, 4: 52-56.

Cuvelier, S, Degrande, J, Merveillie, L, Spruytte, S, Vervaeke, J (2007). Dagvlinders in West-Vlaanderen. Verspreiding en Ecologie 2000-2006, Z.W.V.V.K., Ieper, 144 pp.

Dochy, O, Bauwens, D, Adriaens, T, Vrielynck, S, Maes, D, Decleer, K (2007). Prioritaire en symboolsoorten voor soortbescherming in West-Vlaanderen, Rapporten van het Instituut voor Natuur- en Bosonderzoek Instituut voor Natuur- en Bosonderzoek/Provinciebestuur West-Vlaanderen, Brussel/Brugge, 128 pp.

Maes, D, Vanreusel, W, Van Dyck, H (2007). Vlinderindicatoren. Een handige hulp bij het inventariseren. Natuur.focus, 6: 60-64.

Maes, D, Anselin, A, Decleer, K, De Knijf, G, Fichefet, V (2008). Insecten en klimaatswijziging in België. Van de regen in de drup. Natuur.focus, 7: 107-111.

Beckers, K, Maes, D (2009). De iepenpage in België. In: Heybroek HM, Goudzwaard L, Kaljee H (eds.) Iep of Olm. Karakterboom van de Lage Landen. KNNV Uitgeverij, Zeist, 119-120.

Beckers, K, Ottart, N, Fichefet, V, Godeau, JF, Weyembergh, G, Beck, O, Gryseels, M, Maes, D (2009). Dagvlinders van het Brussels Hoofdstedelijk Gewest: verspreiding, behoud en beheer/Papillons de jour de la Région de Bruxelles-Capitale: répartition, conservation et gestion, Leefmilieu Brussel & Instituut voor Natuur- en Bosonderzoek, Brussel, 158 pp.

Beckers, K, Vermeersch, G, Maes, D, Adriaens, T, De Beer, D, De Knijf, G, Bosmans, R, Hendrickx, F, Jooris, R, Maelfait, JP, Van Den Berge, K, Van Keer, K, Van Landuyt, W, Van Thuyne, G (2010). Een gericht natuurbeleid voor de prioritaire soorten in de provincie Antwerpen. *In:* Dienst Duurzaam Milieu- en Natuurbeleid (ed.) Provinciale Prioritaire Soorten Provincie Antwerpen*.* Instituut voor Natuur- en Bosonderzoek, Antwerpen, Brussel, 13-153.

Van Dyck, H, Maes, D (2010). Zorgwekkende trends voor 'gewone' dagvlinders. Resultaten en lessen na 16 jaar monitoring in de Lage Landen. Natuur.focus, 9: 14-19.

Maes, D, Vanreusel, W, Jacobs, I, Berwaerts, K, Van Dyck, H (2011). Een nieuwe Rode Lijst dagvlinders. De IUCN-criteria toegepast in Vlaanderen. Natuur.focus, 10: 62-71.

Maes, D, Vanreusel, W, Jacobs, I, Berwaerts, K, Van Dyck, H (2011). Nieuwe Vlaamse Rode Lijst dagvlinders. Vlinders, 11: 4-7.

Demolder, H, Peymen, J (2012). Natuurindicatoren 2012. Toestand van de natuur in Vlaanderen: cijfers voor het beleid, Mededelingen van het Instituut voor Natuur- en Bosonderzoek INBO.M.2012.2. Instituut voor Natuur- en Bosonderzoek, Brussel, 44 pp

Adriaens, D, Adriaens, T, De Knijf, G, Hendrickx, F, Maes, D, Van Landuyt, W, Vermeersch, G, Louette, G (2013). Soorten en biotopen in Oost-Vlaanderen: prioriteit en symboolwaarde voor het natuurbeleid, Rapporten van het Instituut voor Natuur- en Bosonderzoek 1040772. Instituut voor Natuur- en Bosonderzoek, Brussel, 387 pp.

Demolder, H, Peymen, J (2013). Natuurindicatoren 2013. Toestand van de natuur in Vlaanderen: cijfers voor het beleid, Mededelingen van het Instituut voor Natuur- en Bosonderzoek INBO.M.2013.1. Instituut voor Natuur- en Bosonderzoek, Brussel, 48 pp.

Herremans, M, Gielen, K (2013). Was 2013 een super vlinderjaar? Cijfers uit losse waarnemingen 2009-2013. Natuur.focus, 12: 154-162.

Maes, D, Vanreusel, W, Van Dyck, H (2013). Dagvlinders in Vlaanderen: nieuwe kennis voor betere actie, Uitgeverij Lannoo nv, Tielt, 542 pp.

Puls, R, Maes, D, Bonte, D (2013). Wordt de grond te warm onder de poten van de Argusvlinder? Is het klimaat verantwoordelijk voor zijn achteruitgang in Vlaanderen? Natuur.focus, 12: 140-144.

Demolder, H, Peymen, J, Anselin, A, Adriaens, T, De Beck, L, Boone, N, De Keersmaeker, L, De Knijf, G, Devos, K, Everaert, J, Jansen, I, Laurijssens, G, Louette, G, Maes, D, Meiresonne, L, Neirynck, J, Simoens, I, Stevens, M, Onkelinx, T, Van Daele, T, Van der Aa, B, Van Landuyt, W, Van Uytvanck, J, Vermeersch, G, Verreycken, H (2014). Natuurindicatoren 2014. Toestand van de natuur in Vlaanderen: cijfers voor het beleid, Mededeling van het Instituut voor Natuur- en Bosonderzoek INBO.M.2014.521558. Mededeling van het Instituut voor Natuur- en Bosonderzoek, Brussel, 53 pp.

Demolder, H, Schneiders, A, Spanhove, T, Maes, D, Van Landuyt, W, Adriaens, T (2014). Hoofdstuk 4 – Toestand biodiversiteit. *In:* Stevens M (ed.) Natuurrapport - Toestand en trends van de ecosystemen en ecosysteemdiensten*.* Mededelingen van het Instituut voor Natuur- en Bosonderzoek INBO.M.2014.1988582. Instituut voor Natuur- en Bosonderzoek, Brussel, 81 pp.

Jacobs, I, Segers, N, Vanreusel, W, Van Dyck, H, Maes, D (2014). Wetenschappelijk basisrapport voor het Soortbeschermingsprogramma Bruine eikenpage (*Satyrium ilicis*), Rapport van het Instituut voor Natuur- en Bosonderzoek INBO.R.2014.1494759. Instituut voor Natuur- en Bosonderzoek, Brussel, 190 pp.

Maes, D (2014). Blauwdruk Vlinders. In: De Knijf G, Westra T, Onkelinx T, Quataert P, Pollet M (eds.) Monitoring Natura 2000-soorten en overige soorten prioritair voor het Vlaams beleid. Blauwdrukken soortenmonitoring in Vlaanderen. Instituut voor Natuur- en Bosonderzoek, Brussel, 170-187.

Segers, N, Jacobs, I, Vanreusel, W, Van Dyck, H, Maes, D (2014). Wetenschappelijk basisrapport voor het Soortbeschermingsprogramma Heivlinder (*Hipparchia semele*), Rapporten van het Instituut voor Natuur- en Bosonderzoek INBO.R.2014.1494593. Instituut voor Natuur- en Bosonderzoek, Brussel, 215 pp.

Segers, N, Van Dyck, H, Jacobs, I, Vanreusel, W, Maes, D (2014). Wetenschappelijk basisrapport voor het Soortbeschermingsprogramma Argusvlinder (*Lasiommata megera*), Rapporten van het Instituut voor Natuur- en Bosonderzoek INBO.R.2014.1494695. Instituut voor Natuur- en Bosonderzoek, Brussel, 106 pp.

Demolder, H, Peymen, J, Adriaens, T, Anselin, A, Belpaire, C, Boone, N, De Beck, L, De Keersmaeker, L, De Knijf, G, Devos, K, Everaert, J, Jansen, I, Lommaert, L, Maes, D, Onkelinx, T, Simoens, I, Stevens, M, Thoonen, M, Van Den Berge, K, Van der Aa, B, Van Gossum, P, Van Landuyt, V, Van Reeth, W, Van Uytvanck, J, Vermeersch, G, Verreycken, H (2015). Natuurindicatoren 2015. Toestand van de natuur in Vlaanderen: cijfers voor het beleid, Mededeling van het Instituut voor Natuur- en Bosonderzoek INBO.M.2015.10366660. Instituut voor Natuur- en Bosonderzoek, Brussel, 63 pp.

Jacobs, I, Segers, N, Vanreusel, W, Laurijssens, G, Van Dyck, H, Maes, D (2015). Nieuwe kennis over de Bruine eikenpage in Vlaanderen. Een ecologische hulpbronnenaanpak voor een bedreigde bosrandsoort. Natuur.focus, 14: 52-57.

Maes, D, De Bruyn, L, De Knijf, G, Onkelinx, T, Piesschaert, F, Pollet, M, Truyens, P, Van Calster, H, Westra, T, Quataert, P (2015). Monitoringsprotocol vlinders, Rapporten van het Instituut voor Natuur- en Bosonderzoek INBO.R.2015.7827697. Instituut voor Natuur- en Bosonderzoek, Brussel, 35 pp.
